# Military Tract Medical Association

**Published:** 1867-07

**Authors:** H. Nance, S. K. Crawford

**Affiliations:** President; Secretary


### MILITARY TRACT MEDICAL ASSOCIATION.

Association met pursuant to adjournment, at Monmouth, May 14th, 1867, 10 A.M., in the Eccrittean Hall of Monmouth College. Meeting called to order by the President, Dr. A. H. Thompson, of Princeton. Opened with prayer by Rev. D. A.

Wallace, D.D., President of the College. Minutes of previous meeting read and adopted.

Election of officers for the ensuing year being next in order, the following named gentlemen were declared officers of the Association: H. Nance, M.D., Kewanee, President; David McDill, M.D., Biggsville, Vice President; S. K. Crawford, M.D., Monmouth, Secretary and Treasurer; A. H. Thompson, M.D., Princeton, J. R. Webster, M.D., Monmouth, and J. C. Smiley, M.D., of Kewanee, Censors.

Dr. Thompson, the retiring President, made some very appropriate remarks on the history and progress of the Association, its present flourishing condition, and prognosticated its future career to be a glorious and successful one.

Dr. Nance, on taking the Chair, thanked the Association for the honors conferred, and proceeded at once to the transaction of business.

Essays being called for, Dr. Webster read one on Cholera Infantum. He recommends the chalk mixture, with the vegetable astringents, after the secretions have been corrected by mercurials, and nourishes his little patients with milk and animal broths, and ice cream and stimulates. The Society, by an unanimous vote, thanked the author of the essay, and recommended its publication.

Dr. Thompson made a verbal report of several cases of vesico-vaginal fistula, upon which he had recently operated, and exhibited instruments.

Dr. Ruce, of Knox, exhibited a calculus of the mulberry variety, taken from a little boy four years old. Dr. Ruce removed it by the ordinary lateral operation. Speedy recovery.

Dr. Webster reported a case of fracture of spinous process of twelfth dorsal vertebra. Rest; recovery.

Dr. Hamilton, of Galesburg, Chairman of Committee on Surgery, read reports from Spaulding, of Galesburg, and Crossley, of Princeton.

Dr. Spaulding thought there was too much conservative surgery practiced, and that the knife is often withheld when its prompt use might and would save the life of the patient.

Dr. Crossley spoke of bromide of potass. in from ten to thirty grain doses, in spermatorrhœa, with the happiest results. Thinks, perhaps, the iodide would answer the same good purpose.

Dr. Hamilton remembered a case of spermatorrhœa in which local cauterization was had recourse to after all else had failed, and was eminently successful.

Dr. Hamilton reported on the use of bromine in erysipelas and gangrene, and thinks, after a fair trial of it, in both civil and military practice, he can give a favorable opinion of its efficacy in those affections for which it has hitherto been so highly recommended. Thinks that bromide of potass. may prove an antidote in hydrophobia.

Drs. Thompson, Ruce, McDill, and others, spoke in laudatory terms of bromine, bromide of potass., and bromide of ammonium.

Dr. Ewing, of Monmouth, Chairman of Committee on Materia Medica and Therapeutics, read reports from Breed and Smiley.

Dr. Breed spoke of new remedies, and prominent among which was bromine and bromide of potass.

Dr. Smiley dwelt at some length on the importance of food, sleep, pure air, and cleanliness, in the management of disease in whatever form.

Dr. Ewing's personal report was not heard, on account of the late hour and amount of other business to be disposed of.

Dr. H. Nance read an able report on obstetrics.

Moved by Dr. Thompson, that in appointing the Committees on different branches of medicine and surgery, it is desired that the reports be all sent to the Chairman of the Committee, and by him condensed and reported to the Association, and that the facts, so far as possible, shall be original, in their own experience, or new as to the literature of the profession. Unanimously adopted.

Drs. Phillips, Spalding, Gilbert, Young, and others, discussed the change of type of disease. Other animated discussions followed, on various topics, and all the members manifested a lively interest.

Moved by Dr. Young, and adopted, that hereafter the Association, at its semi-annual meetings remain in session two days instead of one, as heretofore.

Moved by Dr. Gilbert, and and adopted, that puerperal convulsions and aphasia be the special subjects for consideration and discussion at the next meeting of the Association.

Moved by Dr. Tnompson, and carried, that the next meeting be held in Princeton.

Society adjourned to meet in Princeton on the second Tuesday in December, 1867.

H. NANCE, M.D., President.

S. K. CRAWFORD, M.D., Secretary.

